# RETRACTION: Long noncoding RNA UCA1 promotes cell growth, migration, and invasion by targeting miR‐143‐3p in oral squamous cell carcinoma

**DOI:** 10.1002/cam4.7338

**Published:** 2024-05-30

## Abstract

**RETRACTION:** Q. Duan, M. Xu, M. Wu, X. Zhang, M. Gan, H. Jiang, “Long noncoding RNA UCA1 promotes cell growth, migration, and invasion by targeting miR‐143‐3p in oral squamous cell carcinoma,” *Cancer Medicine* 9, no. 9 (2020): 3115–3129, https://doi.org/10.1002/cam4.2808.

The above article, published online on March 4, 2020, in Wiley Online Library (wileyonlinelibrary.com), has been retracted by agreement between the journal Editor‐in‐Chief, Stephen Tait, and John Wiley and Sons Ltd. Following publication, concerns were raised by third parties regarding the origin of figures 7A and 7C. Unexpected similarities between an instrument in this figure and figures from other papers by unrelated author groups were found. Furthermore, duplication of a panel from figure 6D (showing MK1 cells under control conditions at 24 hours) were found within a figure from another article published elsewhere in a different scientific context. The authors were contacted and invited to comment on the concerns raised and to provide the original, unmodified figures, but did not respond. The editors have lost confidence in the data and conclusions of this study.

**RETRACTION:** Q. Duan, M. Xu, M. Wu, X. Zhang, M. Gan, H. Jiang, “Long noncoding RNA UCA1 promotes cell growth, migration, and invasion by targeting miR‐143‐3p in oral squamous cell carcinoma,” *Cancer Medicine* 9, no. 9 (2020): 3115–3129, https://doi.org/10.1002/cam4.2808.

The above article, published online on March 4, 2020, in Wiley Online Library (wileyonlinelibrary.com), has been retracted by agreement between the journal Editor‐in‐Chief, Stephen Tait, and John Wiley and Sons Ltd. Following publication, concerns were raised by third parties regarding the origin of figures 7A and 7C. Unexpected similarities between an instrument in this figure and figures from other papers by unrelated author groups were found. Furthermore, duplication of a panel from figure 6D (showing MK1 cells under control conditions at 24 hours) were found within a figure from another article published elsewhere in a different scientific context. The authors were contacted and invited to comment on the concerns raised and to provide the original, unmodified figures, but did not respond. The editors have lost confidence in the data and conclusions of this study.

